# Supplementary material for: Extremely cold ocean temperatures in iron formation brine pools of snowball Earth
Source: Nat Commun. 2025 Dec 9;17:462. doi: 10.1038/s41467-025-67155-z (PMC12800124; doi:10.1038/s41467-025-67155-z)
Supplement: Supplementary file 1 — Supplementary Information [file 41467_2025_67155_MOESM1_ESM.pdf]

Supplementary Information for:

Extremely cold ocean temperatures in iron formation brine pools of snowball Earth

Kai Lu, Lianjun Feng, Ross N. Mitchell*, Maxwell A. Lechte, and Paul F. Hoffman

This file includes:

Supplementary Figures 1–4

Supplementary references

Supplementary Data 1 (included as a separate spreadsheet)

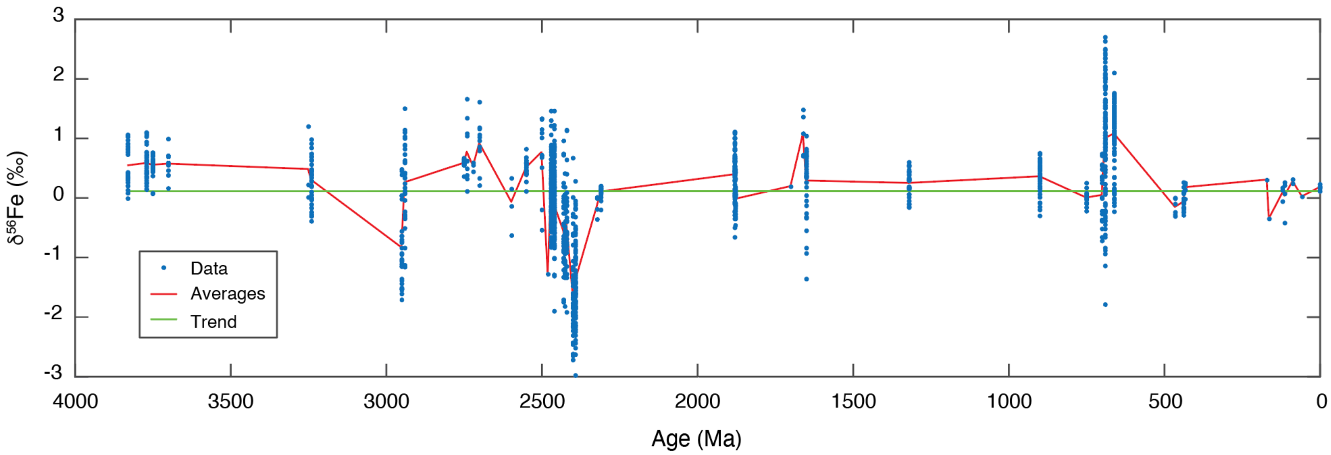

Supplementary Fig. 1 | Secular trend analysis as a test of changes in Fe sources in the ocean. Raw data are blue dots. Red line is age-binned (by average) data such that individual ages with more/less data are not over-/under-weighted. Green line is a linear trend (with free slope) that indicates essentially no secular change (that is, effectively zero slope, where $m = 5 \times 10^{-14}$).

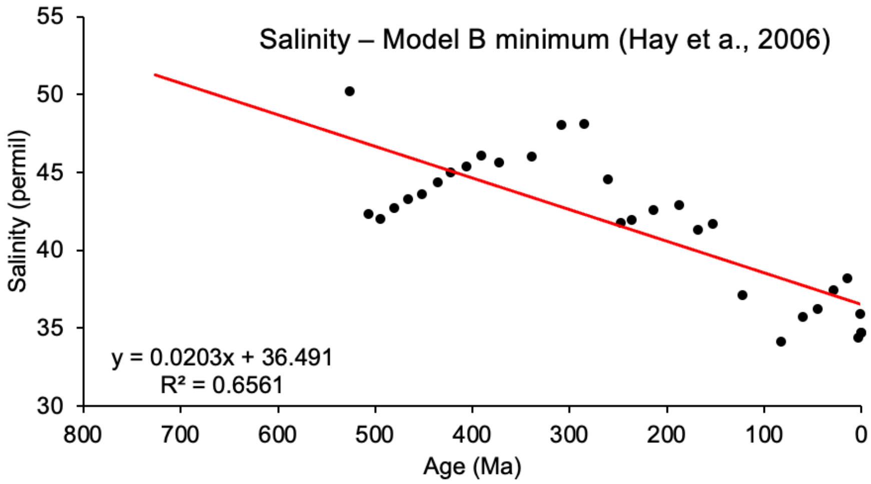

Supplementary Fig. 2 | Salinity extrapolated from the Phanerozoic to the Cryogenian. The minimum estimate of Model B of ref. [1] was used as a conservative estimate of salinity. Model B assumes constant ocean volume over time. At 676 Ma, the middle of the Cryogenian, salinity is estimated to be 50.2‰ (or PSU).

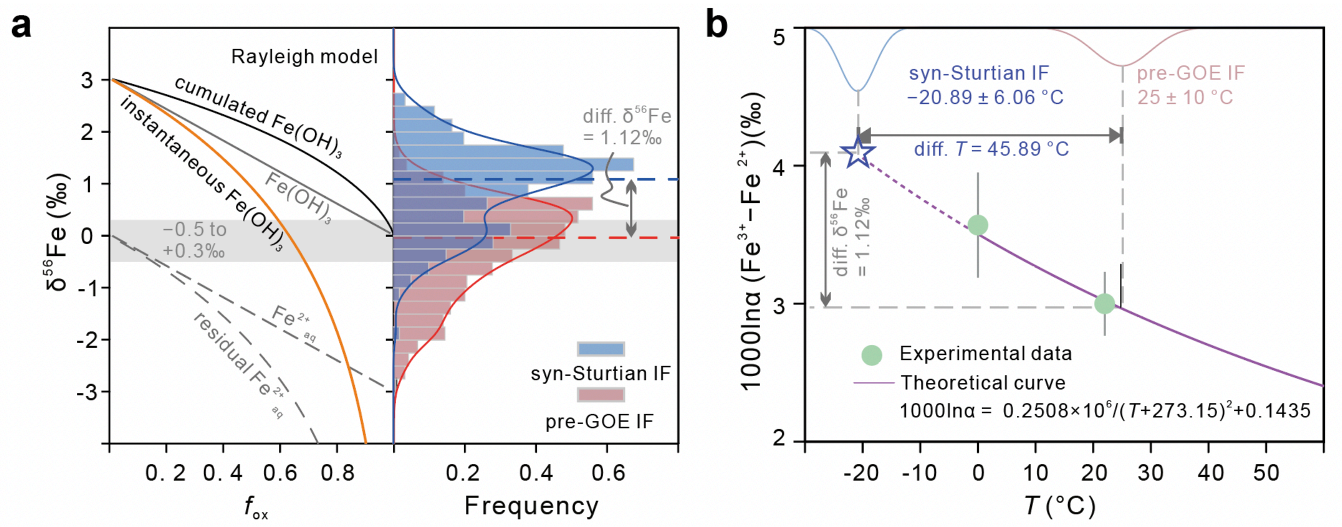

Supplementary Fig. 3 | Temperature estimates of snowball Earth according to the CIF δ^{56}Fe anomaly including the Fulu Fm. Using the same method as Figure 3, except including data from the Fulu Fm, a colder temperature of −20.9 ˚C is estimated for the snowball ocean. The more conservative estimate presented in the main text (excluding Fulu Fm) is preferred given both the age ambiguity of the Fulu Fm and its lack of direct evidence for glaciation[2].

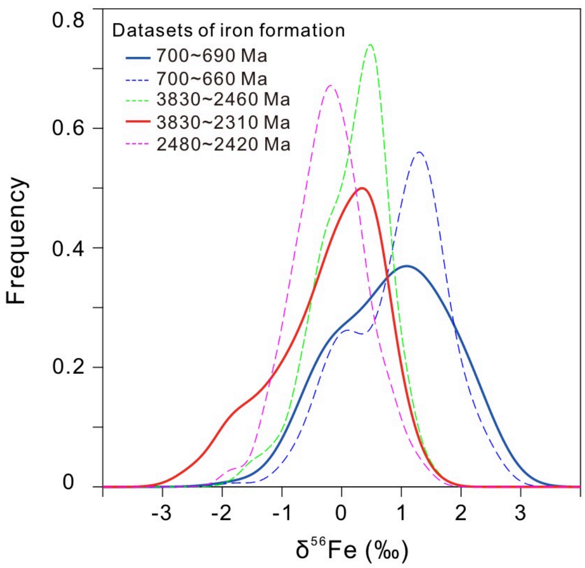

Supplementary Fig. 4 | Distributions of δ^{56}Fe datasets being compared. Distribution of δ^{56}Fe values for various Cryogenian and pre-GOE time intervals. The solid lines (blue and red) were ultimately used for comparison because of their similar range and statistical distribution.

Supplementary Data 1. Fe isotope data (included as a xls file)

Supplementary references

1 Hay, W. W. *et al.* Evaporites and the salinity of the ocean during the Phanerozoic: Implications for climate, ocean circulation and life. *Palaeogeography, Palaeoclimatology, Palaeoecology* **240**, 3-46 (2006). https://doi.org/https://doi.org/10.1016/j.palaeo.2006.03.044

2 Wu, C.-Z. *et al.* Genesis of the Fulu Cryogenian iron formation in South China: Synglacial or interglacial? *Precambrian Research* **376**, 106689 (2022). https://doi.org/https://doi.org/10.1016/j.precamres.2022.106689
