# Supplementary material for: Extremely cold ocean temperatures in iron formation brine pools of snowball Earth
Source: Nat Commun. 2025 Dec 9;17:462. doi: 10.1038/s41467-025-67155-z (PMC12800124; doi:10.1038/s41467-025-67155-z)
Supplement: Supplementary file 2 — Description of Additional Supplementary Files [file 41467_2025_67155_MOESM2_ESM.pdf]

Description of Additional Supplementary Files:

Supplementary Data 1: Fe isotope data
